# Design and measurement in a study of war exposure, health, and aging: protocol for the Vietnam health and aging study

**DOI:** 10.1186/s12889-019-7680-6

**Published:** 2019-10-23

**Authors:** Kim Korinek, Bussarawan Teerawichitchainan, Zachary Zimmer, Eleanor Brindle, Thi Kim Chuc Nguyen, Huu Minh Nguyen, Khanh Toan Tran

**Affiliations:** 1 Department of Sociology, University of Utah, 380 S 1530 E, Room 301, Salt Lake City, Utah USA; 2 National University of Singapore, Singapore, Singapore; 3 Mount Saint Vincent University, Halifax, Canada; 4 University of Washington, Seattle, USA; 5 Hanoi Medical University, Hanoi, Vietnam; 6 Vietnam Institute for Family and Gender Studies, Hanoi, Vietnam

**Keywords:** Study protocol, Aging, Stress, Post traumatic stress, Armed conflict, Asians, Vietnam, Survey research, Field-based biomarker collection

## Abstract

**Background:**

Survivors of war throughout the world experience illnesses and injuries that are crucial to understand, given the ongoing treatment and adaptation they demand. In developing countries like Vietnam, where population aging and chronic disease burdens are rapidly rising, aging populations have seen a disproportionate share of armed conflict and related casualties. This paper describes the Vietnam Health and Aging Study (VHAS), a unique resource for investigating mechanisms of association between diverse exposures to armed conflict during the Vietnam War and multiple dimensions of older adult health among survivors of that war.

**Methods:**

The VHAS utilizes a longitudinal design, the first wave of data collection conducted in 2018 among 2447 older adults. A second wave of follow-up data collection, scheduled to take place in 2021, will examine life course, social relational and health and mortality transitions. The VHAS was conducted in four northern Vietnamese districts purposively selected to represent a spectrum of war exposure as indicated by intensity of bombings. Additionally, VHAS uses random sampling within gender and military service subdomains to permit unique gender-specific analyses of military service, trauma exposure and health.

The VHAS’ face-to-face interviews include modules detailing war and military service experiences; warzone stressors; and multiple dimensions of health such as chronic disease, functional limitation, disability, health behaviors, cognition and psychological health. Biomarker data collected for the full VHAS sample includes anthropometric and functional tests such as grip strength and blood pressure, hair samples for cortisol assay, and capillary blood samples to assay C-reactive protein, cholesterol, HbA1c, and other markers of interest for cardiovascular and other disease risks and for testing the impact of early life stressors on later life health. Blood samples will also permit epigenetic analysis of biological aging.

**Discussion:**

Future VHAS investigations will examine dynamic linkages between war exposure, mortality and morbidity, while taking into account the selective nature of each of these processes. Longitudinal analyses will examine late-life health transitions and war-related resiliency.

## Background

Survivors of war live throughout the world and their numbers are increasing. Their illnesses and injuries are arguably as challenging to societies as war deaths and as crucial to understand, given the ongoing treatment and adaptation they demand [1–9]. It is therefore incumbent upon us to understand and address war’s long-term impact on the course of aging, global disease burdens, and demands upon public health and support systems.

Late 20th and early twenty-first century wars, as well as related post-war recovery and readjustment challenges, have concentrated in low-middle income countries where war-damaged institutions and infrastructure layer upon fragile economies, unstable political systems, and weakly developed healthcare systems [10]. Yet, populations and members of armed forces from the global south are largely absent from the base of evidence linking military service and war trauma to later life health outcomes [11–13]. Extant research focusing upon American male service members, especially those exposed to combat, demonstrates marked influences on health, mobility and family relations, for instance heightened risks of PTSD, smoking and subsequent heart disease and lung cancer, marital discord and divorce [14–18]. Studying war survivors, among whom exposures ranged from mild to extreme, and whose modes of war participation ranged from formal to informal and active to passive, allows for examination of groups most susceptible to long-term impacts from war exposures earlier in life. This knowledge has theoretical and practical value across societies of the global south where exposure to violent armed conflict is widespread and enduring. These enduring influences of war are likely to be especially critical as men and women age and as chronic disease and disablement processes begin to accelerate.

Due to protracted military conflicts with the French and Americans over much of the twentieth century, Vietnam is a critical context for furthering knowledge of war’s lasting public health impacts. The Vietnam War (known among Vietnamese study participants as the “American War”) exacted a severe toll upon Vietnamese society [19, 20], yet knowledge of its enduring impacts still derives largely from American soldiers’ perspectives [21, 22]. Indeed, while several studies focused upon US veterans show that the long-term impacts of war are consequential for aging populations (as evidenced by a recent special issue of *The Gerontologist* on Veterans Aging [23–26]), knowledge is strikingly sparse and geographically narrow, deriving mostly from the West, and two groups in particular: US veterans and war refugees [27–31]. Research in the developed world tells us that veterans and war refugees face enhanced risks of physical and mental health problems, from heart disease to post-traumatic stress disorder, diabetes and other chronic diseases [32–38]. In developing countries like Vietnam, where population aging and chronic disease burdens are rapidly rising, aging populations have seen a disproportionate share of armed conflict and related casualties. Yet, aside from scholarship focused on the effects of Agent Orange exposure, war’s enduring population-level impacts in these contexts remain greatly understudied [39, 40].

The Vietnam Health and Aging Study (VHAS) is a collaborative research study allowing for investigation of the mechanisms of association between diverse exposures to armed conflict during the Vietnam War and multiple dimensions of health among older adults. The VHAS will provide a unique, publicly available data resource for methodologically rigorous exploration of the implications of armed conflict for older adult health and aging processes. The rich combination of health and war exposure measurements are unprecedented and allow analysis of the dynamic linkages between war exposure, mortality, and morbidity, while taking into account the selective nature of each of these processes. The VHAS also equips investigators to understand how family relationships, stressful experiences in the life course, and current material, social and stressor conditions serve as mediating factors between wartime experiences, aging processes and late life health outcomes.

Funded by a grant from the National Institutes of Health/National Institute on Aging (R01 AG052537), the VHAS is conducted by an interdisciplinary team of social scientists and health scientists from the University of Utah, USA; Mount Saint Vincent University, Canada; the National University of Singapore; University of Washington, Seattle, USA; Hanoi Medical University, Vietnam; and the Institute for Family and Gender Studies, Vietnam.

## Methods

### Overview

The VHAS utilizes a longitudinal design in order to examine dynamics in the life course, health transitions of older adults, and the developmental processes of resiliency and scarring linked to early life war exposures. Aspects of sampling and the interview components are geared toward documenting the wide-ranging severity and forms of war exposure that characterize this cohort of older Vietnamese adults. The first wave of VHAS data collection was conducted between May and August 2018, among 2447 men and women born in 1959 or earlier (i.e., currently ages 60 and older). A second wave of follow-up data collection, scheduled to take place in 2021, will revisit original respondents in order to ascertain mortality and, among survivors, changes in health behaviors and health conditions, socioeconomic conditions, living arrangements, intergenerational and social relationships. Both waves of data collection include face-to-face, home-based interviews, conducted by staff members of Hanoi Medical University and the Institute for Family and Gender Studies, and biomarker measurement conducted by staff members of Hanoi Medical University in local commune health centers. Senior personnel from all six participating academic institutions trained and supervised the interviewer and biomarker data collection teams.

### Sampling

**Fig. 1:**
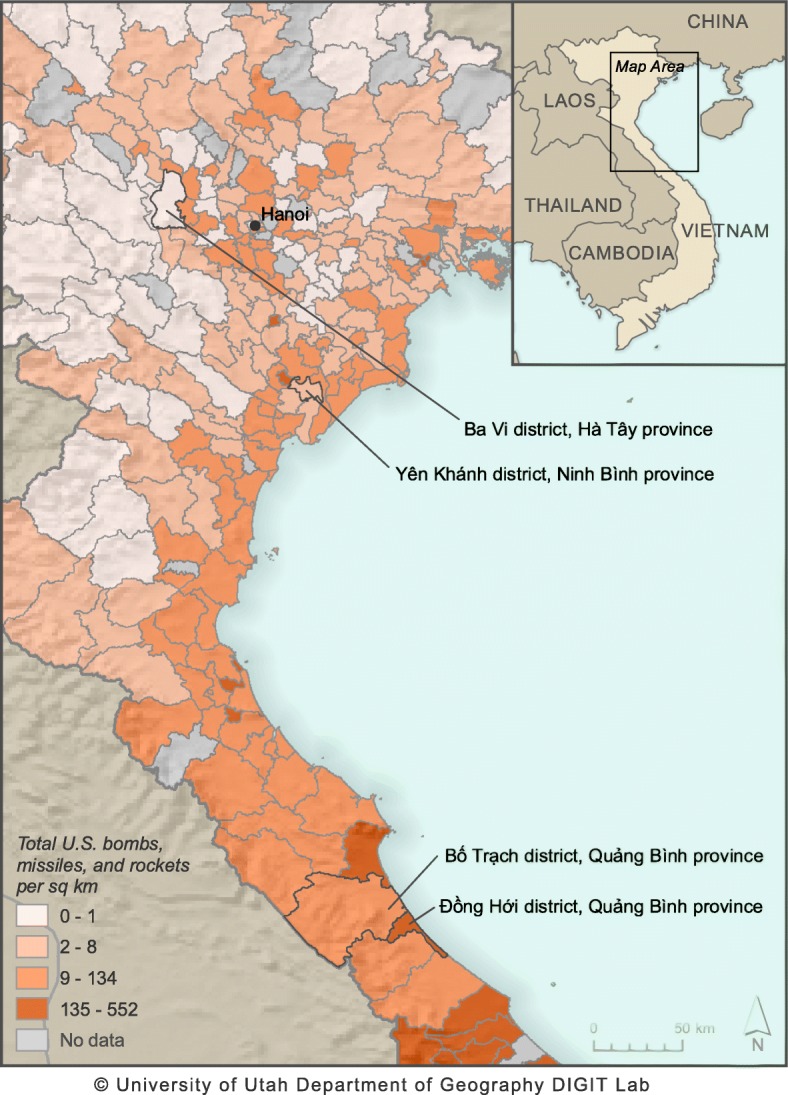
Map Delineating Districts for VHAS sampling. Map created by Digit Lab, University of Utah (https://digit.utah.edu/).© University of Utah Department of Geography DIGIT Lab. Districts are categorized according to exposure to all ordnance dropped from U.S. and allied airplanes and helicopters in Vietnam between 1965 and 1975, as well as artillery fired from naval ships. These data come from the 1965–70 Combat Activities-Air (CACTA), the 1970–75 Southeast Asia (SEADAB), and Combat Naval Gunfire (CONGA) databases

The VHAS relied upon a multistage probability design to arrive at the study sample. We began by purposively selecting four districts in northern Vietnam, which represent a spectrum of exposures to war as indicated by the intensity of bombings during the 1960s and 1970s [41]. Specifically, Ba Vi district within Ha Noi province, Yen Khanh district within Ninh Binh province, and Bo Trach district and Dong Hoi city within Quang Binh province, represent low, moderate and high bombing intensities, respectively (See Fig. 1). To arrive at our Primary Sampling Units (PSUs) we randomly selected sub-geographic units (communes in rural areas, wards in urban areas) in each district as follows: four communes within Ba Vi district (Hanoi); four communes within Yen Khanh district (Ninh Binh); two communes within Bo Trach district and two wards within Dong Hoi city (Quang Binh). These 12 communes and wards constitute the PSUs for VHAS sampling.

To create our sampling frame of eligible respondents within each PSU (i.e., current, community-dwelling residents age 60 and older) the VHAS team collaborated with local officials to access current, regularly updated household registration databases. In particular, VHAS staff sought the assistance of population volunteers (cộng tác viên dân sộ) to derive the sampling frame. Population volunteers are members of the community tasked with liaising with local governments regarding population-related matters within their village/ward, such as population enumeration and routine reporting of births and deaths. The population volunteers utilized local record-keeping systems to generate a list of all households with at least one member age 60 or older. From these households we delineated the following information among potential respondents: (a) address, (b) name, (c) age, (d) sex, and (e) veteran status. If local officials lacked information about the individual characteristics (b)-(e), they contacted households via phone or home visit to confirm the information. A full listing of characteristics a) - e) was created for all persons 60 and older in each commune/ward, thus comprising our sampling frame. If more than one person in the household was age 60 or older, both members were included in the listing. “Veteran status” for the purpose of our study includes participation in both formal military roles or as voluntary participants in militia/paramilitary units such as the Youth Shock Brigades (*Thanh Niên Xung Phong*). The latter, a military corps established in 1950 and widely reactivated by Democratic Republic of Vietnam during 1965–1975, recruited more youthful, teenage volunteers, including many young women, into military support roles such as defusing bombs, maintaining roads, and provisioning arms and food to the troops [42]. Like formal service members, members of the Youth Shock Brigades also often encountered the extreme conditions of the frontline (Ibid).

The VHAS is the first comprehensive population-based survey in Vietnam to examine health outcomes as they vary across forms of war exposure. The VHAS considers a large number of factors for analysis and therefore a single, formal sample size calculation for the entire study is not feasible. However, we use several criteria to evaluate a sample size that has generally suitable power. First, using the PIs’ pilot study, the Vietnam Health and Aging Pilot Study conducted in 2010 among 405 Vietnamese older adults, we estimated prevalence rates for several outcomes of interest (e.g., PTSD symptoms, arthritis, heart disease, and others) as they varied across men and women who were differently exposed to the “treatment” effect of military service during the Vietnam War [43]. For example, based on the pilot study, we observed the following heart disease prevalence rates across male and female veterans, and male and female nonveterans, respectively: 13.3, 20.0, 10.3 and 16.7%. For the same gender-veteran status categories, we observed the following prevalence rates for disabling arthritis: 38, 20, 75 and 52.8%. We use these estimated prevalence rates, an expected response rate of 90% (as observed in the pilot study and other population-based surveys in Vietnam), and an anticipated 20% attrition across waves of the survey, to arrive at sample size of 2497 necessary to achieve statistical power greater than 80% for a .05 level of significance [44]. This sample size was determined based upon the statistical power calculation for the heart disease outcome, whereas current PTSD and disabling arthritis, both more prevalent and more sharply differentiated across veterans and nonveterans in the pilot study, suggested an estimated sample size of between 345 and 450 persons to achieve the same degree of statistical power.

In addition to conventional statistical power calculations, we also consulted Peduzzi et al’s ‘rule of thumb’ for a minimum number of ‘events per variable,’ [45] to arrive at subdomain sample sizes that allow for stable multivariate regression analyses of outcomes of interest. Following this logic, we arrived at a similar sample size to the statistical power calculation. Specifically, the Peduzzi method suggested a total target sample size of 2448, or approximately 204 persons per PSU. Gender and veteran status served as our key screening questions [46] and allowed us to organize and place all potential study participants within each PSU into four subdomains: male-veteran, male-nonveteran, female-veteran, and female-nonveteran. Within each PSU, we listed potential participants’ names according to village of residence in order to ensure a geographically diverse sample within the commune/ward. Sampling rates varied across subdomains, depending upon the relative size of the eligible population. Individuals were selected from the sampling frame using a stratified systematic, random approach. We sampled randomly within each subdomain/strata in order to arrive at a minimum subdomain sample size to achieve adequate statistical power to conduct theoretically driven analyses of gender and wartime exposure.

As noted below, due to minor difficulties in identifying eligible individuals for inclusion in the sampling frame and a few refusals during in-progress interviews, the final VHAS sample size is 2447. With the application of sampling weights, VHAS data are representative of the older adult population ages 60 and older residing in the twelve communes/wards from which the sample was drawn.

### Recruitment of VHAS participants

In order to recruit participants to the study, local population volunteers contacted potential respondents via telephone and in person, described the study, and invited their participation. Population volunteers made up to three attempts to contact the randomly selected eligible participants to invite their participation and schedule an interview. When a person within the sampling list could not be contacted or refused to be interviewed, a replacement was sought from within the same subdomain in that commune’s/ward’s sampling frame list.

Upon agreeing to participate, the VHAS staff scheduled a two-hour appointment with each prospective respondent, specifying that the private interview could be conducted in the participant’s household or at another preferred location, as well as an appointment for an approximately 35 min health exam, which included a series of anthropometric measures, hair and capillary blood collection. The biomarker collection appointment was scheduled to take place within the commune health center (CHC) one or 2 days after the interview. The VHAS staff also provided those who agreed to participate with an appointment reminder and a copy of the study consent form so that they could become more familiar with the study procedures and their rights as human subjects in advance of the team’s reviewing the consent form and obtaining their informed consent at the start of the interview.

As part of the recruitment and study invitation, each prospective respondent was informed that they would be paid an incentive payment of 90,000 Vietnamese Dong, approximately $US 4.50, for their participation in the study. In addition, VHAS staff cooperated with local health clinic staff to provide a general health examination and consultation alongside the biomarker collection exam.

The project team offered to assist with prospective respondents’ transportation to the CHC if the individual was unable to travel on their own or with family assistance. In the case that a respondent was unable to travel to the CHC, even with assistance, the VHAS staff arranged for a clinic staff member to make an in-home visit to collect the biomarker measures, excluding the collection of blood, per Ministry of Health policy. Additionally, for persons who missed their scheduled biomarker collection appointment, the VHAS staff followed up and offered an in-home visit for collection.

### Response rates

Numbers and rates of nonresponse were calculated for the total VHAS sample, for each of the 12 PSUs (communes and wards), and within-PSU, for each of our four study subdomains. Nonresponse was due to refusal to participate, lack of availability to participate, or inability of project staff to locate the enumerated individual. The overall response rate for the VHAS Wave I data collection is 84.7%, ranging from 81.0 to 93.2% across PSUs. In terms of the study subdomains, those in the female veteran domain had the highest response rate (91.2%), while the female nonveteran domain had the lowest response rate (81.7%). The male veteran and nonveteran domain response rates were intermediate: 86.2 and 85.8%, respectively. The refusal rate, i.e., the percentage of those selected for study participation who refused to participate, was 9.7% overall (ranging across communes from 2.5 to 16.7%).

For individuals selected from the frame, but who did not participate due to refusal or other reasons, a replacement was randomly selected from within the same gender and veteran status subdomain for that ward/commune. There are 458 of such cases. Apart from refusing to participate in the survey, another main reason for case replacement is temporary migration (e.g., to visit distant children/grandchildren) during the time the survey was taking place.

### Details of VHAS data collection

#### Study setting

The VHAS Wave I data collection was conducted over 4 months during 2018. The dates for data collection were as follows: Bo Trach district and Dong Hoi city, Quang Binh Province: May 7 to June 1; Ba Vi district, Ha Noi city, June 5–16 and July 2–17; Yen Khanh district, Ninh Binh Province: July 25–August 21.

Interviews were conducted privately in the homes of participants. The interview began by introducing the research project and obtaining informed consent of individuals to participate in the study. After informed consent was obtained, face-to-face interviews were conducted using the Computer Assisted Personal Interviewing (CAPI) system, COMMCARE. Each interviewer utilized a tablet PC to enter data as they conducted the interview. The use of CAPI enhanced the detection and correction of on-the-spot data entry errors. When the interviewer entered an answer with a logic error or abnormal value, the system showed a prompt to caution the interviewer. Interviewers uploaded the data to the data server after each day’s data collection. The average duration of each in-home interview was approximately 2.5 h. Given this duration, interviewers were advised to attend to the comfort, attention and fatigue of the participants. They offered a break when it appeared necessary, or approximately half way through the interview.

#### Proxy interviews

If a sampled respondent was too frail to answer the entirety of the interview questions, we identified a proxy respondent to either fully complete or assist in the completion of the interview. Proxy respondents tend to be the spouse or adult child of the respondent, knowledgeable about his/her life history and familiar with his/her current health and living conditions.

The need for a proxy respondent for the full interview was determined at the study invitation stage, or by the interviewer at the beginning of the interview. Where necessary, such as in cases of ambiguous cognitive functioning, interviewers sought the guidance and approval of field supervisors to make the determination. Once this was determined, the CAPI system automatically switched to the substitution mode before entering the first section. Compared to the normal mode, the proxy respondent was asked only a subset of questions. Proxy respondents answered only factual questions, not subjective questions such as self-rated health, feelings of depression, or marital satisfaction. At the end of each section as well as at the end of the entire interview, interviewers made an assessment about the extent to which proxy respondents assisted in the interview. Possible responses included (1) sampled respondent answered all questions him/herself; (2) sampled respondent received some assistance from others who happened to be present; (3) sampled respondent was somewhat incapable of answering the questions and thus received assistance; (4) proxy respondent answered all of the questions.

As shown in Table 1, the overall share of interviews in which a proxy completed the entire interview was 3.2% (a partial proxy assisted with interviews in an additional 5.5% of interviews). The rate of proxy interview was higher for women than men and was highest for women age 80 and older, reflecting this group’s greater likelihood of experiencing disability or cognitive decline relative to younger participants.

**Table 1:** Proxy rate by age and gender

	Age group	Total Interviews		Respondent Interview		Proxy Interview	
		Freq	% Dist	Freq	Interview	Freq	Proxy
					rate (%)		rate (%)
Total	All	2445	100	2366	96.8	79	3.2
	60–69^a^	1349	55.2	1388	99.2	11	0.8
	70–79	693	28.3	672	97	21	3
	80+	403	16.5	356	88.3	47	11.7
Male	All	1194	100	1162	97.3	32	2.7
	60–69	687	57.5	680	99	7	1
	70–79	335	28.1	325	97	10	3
	80+	172	14.1	157	91.3	15	8.7
Female	All	1251	100	1204	96.2	47	3.8
	60–69	662	52.9	658	99.4	4	0.6
	70–79	358	28.6	347	96.9	11	3.1
	80+	231	18.5	199	86.2	32	13.9

^*a*^ *Note that 2 respondents were actually 59 years of age at the time of Wave I data collection*

#### Components of the VHAS interview

The VHAS survey covers subject areas that allow for testing hypotheses derived from the project’s conceptual framework as well as areas that touch on matters of common interest in the study of aging populations. Specifically, survey modules include the following:

- A household roster, including demographic characteristics of the respondent and all household members;
- In-depth description of military service (formal military and paramilitary);
- War era experiences and stressors associated with warzone activity, including stressors experienced by combatants and noncombatants, in battle and in civilian life;
- Self-reports of myriad dimensions of health, including chronic disease, functional limitation, disability, health behaviors, cognitive and psychological health;
- Rosters of all siblings and children, those living and deceased;
- Co-resident and non-co-resident children characteristics, including intergenerational support and relations;
- Individual and household socioeconomic details such as wealth and education, sources of income, health insurance and other benefits, and employment history;
- Migration history, focused upon mobility during the war era;
- Family background information, childhood health conditions;
- Religion and practice of religion;
- Stressful life events and stress appraisal;
- Social engagement and social support relationships.

### VHAS biomarker data collection

#### Overview

Biomarker data collection is central to the VHAS assessment of health conditions and aging processes. The study investigators seek to understand how early life stressors experienced during wartime influence health and aging over the life course. Accordingly, the VHAS biomarker collection and assay plan focuses upon measuring the physiological pathways linking traumatic stress, chronic stress, health outcomes and physiological aging. The biomarker data includes blood samples that are to be used to assay markers of interest for cardiovascular and other disease risks pertinent to an aging population and for testing the impact of early life stressors on later life health and physiological aging. Biomarkers of interest include, but are not limited to, C-reactive protein, total and HDL cholesterol, and glycated hemoglobin (HbA1c). Other biomarker data collected include blood pressure, hair samples for the testing of cortisol, anthropometric tests and functional tests such as grip strength.

Overall, participation in the biomarker collection component of the study was high, with a participation rate similar to or better than rates observed in comparable studies such as the China Health and Retirement Longitudinal Study and Health and Aging in Africa Longitudinal Study. 2342 participants (95.7% of the total sample) agreed to participate in the biomarker data collection, and at least one valid body measure or function test data point was collected from 2325 participants. 2288 participants (93.5% of the sample) provided blood samples for point-of-care test(s), and a sample of capillary blood was retained from 2210 participants (90.4%) for subsequent laboratory-based analyses.

#### Biomarker collection procedures

All VHAS participants were asked to visit a CHC near their homes for biomarker data collection. Twelve CHCs hosted biomarker data collection. Trained VHAS staff conducted biomarker data collection in two phases: hair sample collection, body measures and functional tests were conducted in one room or clinic area, and blood sample collection and testing were carried out in a separate space. Where possible, biomarker data collection areas were private, but in some cases the collection procedures were visible from common areas of the clinic. At each study location, two study staff were responsible for collecting hair samples, body measures and functional measures. Blood collection and testing required three study staff. A biomarker supervisor was responsible for overseeing both parts of the biomarker procedure, and for assisting the participants in checking in and moving through the steps of the biomarker data collection process.

For study participants deemed by VHAS interview staff or local CHC staff to be too frail to visit the CHC setting, VHAS staff conducted in-home measurement of a subset of biomarkers. VHAS biomarker staff exercised their best judgement to collect only those physical measures that were not too taxing upon the participant. In accordance with Vietnam’s Ministry of Health policies, hair collection and blood sample collection were only conducted in the clinic setting; no biological samples were collected in the home-based visits.

We list the biomarker variables collected in the VHAS in Table 2. Biomarkers included are limited to those that could be measured using minimally-invasive methods and through the use of limited, portable equipment that could be moved from one CHC to another.

**Table 2:** Summary of Biomarkers and Relevant Sample Size, Wave I, VHAS

Biomarker	*N*
Body measures	
Height	2267
Weight	2270
Percent body fat	2018
Mid-upper arm circumference	2278
Calf circumference	2241
Waist circumference	2272
Hip circumference	2272
Functional measures	
Peak expiratory flow	2250
Grip strength	2251
Blood pressure (systolic, diastolic, pulse)	2323
Point-of-care measures	
Complete Blood Count (CBC)	2207
Glycated hemoglobin (HbA1c)	1964
Samples retained for laboratory testing	
Plasma	2208
Buffy coat (peripheral blood mononuclear cells)	2210
Hair	2215

For the majority of participants, the biomarker testing procedures took approximately 35 min to complete, with approximately 15 additional minutes of wait time.

#### Body measures

Anthropometric measurements included standing height, weight, body fat percent, mid upper arm circumference, calf circumference, hip circumference and waist circumference. Procedures for anthropometry followed methods described in the National Health and Nutrition Examination Survey Anthropometry Procedures Manual [47]. Height was measured using a Seca 213 portable stadiometer. Weight and percent body fat were measured using an Omron HBF-514 body composition monitor and scale that estimates body fat using whole-body bioimpedance analysis. Circumference measures were made using Seca 201 circumference measuring tapes.

#### Functional measures

Peak expiratory flow was measured using a Clement Clarke Airzone peak flow meter, with three measures taken per participant. Grip strength was measured using Charder MG4800 Digital Handgrip Dynamometers, with two measures taken per hand; participants were asked to report their dominant hand. Blood pressure was measured, two times, using Omron BP786N automated blood pressure monitors; systolic and diastolic blood pressure were recorded as well as pulse.

#### Hair sample collection

A section of hair was collected for cortisol analysis. A hair sample approximately the diameter of a pencil was tied with string, and cut as close as possible to the scalp from the posterior vertex of the occipital lobe. Samples were wrapped in foil and stored noting the scalp end of the sample. Cortisol will be tested using a one-centimeter segment of hair closest to the scalp.

#### Capillary blood collection

A phlebotomist on the biomarker team collected blood from a finger puncture with a sterile lancet. Blood was collected into BD Microtainer collection tubes with K2-EDTA anticoagulant. A minimum of 0.4 mL and up to 0.6 mL of whole blood was collected into microtainer tubes from each participant. Within 15 min of collection, blood was centrifuged, the plasma fraction and buffy coat layer were separated, and each retained in cryovials. All samples were frozen within one hour of collection, and most were frozen within 15 min of collection. Samples were stored in a -20C freezer at the CHC for up to 1 week. At the end of data collection in each CHC, specimens were transported on ice to a central facility and frozen at -80C. At the end of data collection in all provinces, all samples were transferred on ice to Hanoi Medical University for long-term storage at -80C. The plasma fraction will be used for testing biomarkers indicative of immune function, metabolism, and cardiovascular disease risk. The buffy coat fraction containing peripheral blood mononuclear cells may be used for genetic analyses (pending funding), including DNA methylation and telomere length. For 80 participants (3.5% of all capillary blood collection attempts), the obtained sample either lacked adequate volume or for other reasons was not adequate for laboratory assays.

#### Point-of-care testing

A QBC STAR Centrifugal Hematology System was used to perform complete blood count. The QBC STAR uses 65 to 75 uL of whole blood collected in a capillary tube containing reagents to differentially stain white blood cells. The instrument separates blood by cell type within the capillary tube by centrifugation then uses optical scanning to quantify the cell fractions. The QBC STAR system has on-board automatic quality control procedures that were performed each day at the start of testing. HbA1c was measured using a Diazyme SMART assay system. The test is a direct enzymatic assay of HbA1c requiring 20uL of whole blood. A single finger puncture was used for both capillary blood sample collection and for blood used in the point-of-care instruments; in some cases, a second finger puncture was required to produce enough blood to complete the full protocol. Blood collection was adequate to carry out at least one point-of-care test in every case but one.

#### Biomarker collection limitations

Capillary blood collection, while less invasive, yields a limited volume of sample. However, collection from a finger puncture was typically successful in producing adequate volume for point-of-care tests and for later testing in a laboratory using multiplex assay methods that allow multiple measures simultaneously in a single test. The most significant biomarker challenge was controlling the temperature and humidity in the room where point-of-care testing was conducted. A portable dehumidifier and a portable air conditioner were positioned near the testing instruments, and temperature and humidity were tracked throughout the day.

Performance of the Diazyme SMART HbA1c instrument was of particular concern given previous reports of difficulty with HbA1c measurement in hot and humid climates [48, 49]. Quality control materials were tested at least once per week using each of the two SMART instruments used in fieldwork, and results were found to be within the expected range stated by the manufacturer. However, HbA1c values were generally higher than expected. During fieldwork, a different HbA1c test was used in parallel with the Diazyme SMART for a small subsample, and agreement between the two methods was poor. Diazyme technical support staff evaluated test parameters from the instruments during fieldwork and reported that the instruments were functioning as expected. After fieldwork was completed, one instrument was shipped back to the U.S. for testing in a controlled climate laboratory at UW. Testing using a quality control set from a different manufacturer (Streck) yielded results within the expected range, but at the high end of the range. The source of the unexpectedly high HbA1c results is unclear, and requires further exploration to determine the best approach for use of these data. Possible explanations include poor performance of the Diazyme SMART with hemoglobin variants common in Southeast Asian populations [50, 51].

## Discussion

To date, data on exposure to war and extreme stress is almost entirely lacking in studies of health and aging in the developing world. The Vietnam Health and Aging Study has created a longitudinal data resource for analyses of war exposure and its long-term impacts on health and wellbeing. The Wave I VHAS data were collected in 2018 through in-person interviews and biomarker data collection among 2447 adults age 60 and older. VHAS study participants are survivors of the Vietnam War, which was waged for over a decade during their adolescence and young adulthood. Analyses of the VHAS cohort’s war-time and subsequent life experiences, current health conditions and health trajectories between Waves I (2018) and II (2021) will allow scholars to address the following broad sets of questions based on both social survey measures and biomarkers:

- Does war exposure exert enduring effects on older adults’ health? What forms of exposure are most influential? Are combat and severe war traumas associated with distinct mortality and morbidity patterns in later stages of the life course?
- How do socioeconomic conditions, stress experiences and responses, and social relational factors mediate and moderate relationships linking war exposure and old-age health?
- Does war exposure influence late-life health transitions? Does the onset of disease vary by forms and degrees of wartime exposure? Is war-related resiliency implicated in health transitions?

Alongside these broad lines of inquiry, a broader aim of the project investigators is to open a new field of inquiry on the global demographic and health consequences of armed conflict.

The VHAS has both strengths and limitations. As an omnibus survey of health and aging, the VHAS provides a holistic picture of participants’ material, social, familial and health conditions. A notable strength of the VHAS is the one-of-a-kind, in-depth information that it provides on wartime experiences, as well as rich, detailed information on physical, mental, and functional health status. Yet taking into consideration budget constraints and respondent burden, there are domains for which we would have liked to collect information that is more detailed. Particularly salient issues that we wish we knew more about, such as experiences with economic stress, cognitive decline, and chronic and daily life stressors, will be considered for inclusion in the VHAS Wave II data collection.

As is the case with any population-based study initiated among older adults, samples are necessarily impacted by selective survivorship. This is particularly true in a setting such as Vietnam that has experienced much war-induced premature mortality. The VHAS provides exhaustive information on participants’ sibships. This information can be leveraged to discern differential survivorship across and within families and thereby to ascertain the social dimensions of selective survivorship.

Data collection for the VHAS was influenced by periods of very high heat and humidity. The VHAS study team undertook many efforts, such as implementing early morning scheduling and portable cooling systems, in attempts to maintain suitable climatic conditions for conducting interviews in the homes of elderly individuals and blood collection in CHCs that lacked air conditioning. VHAS interviewers were attentive to and addressed older adults’ levels of comfort during the interview. However the lengthy interview (approximately 2.5 h on average), coupled with the high heat, resulted in fatigue for certain participants, especially the oldest among them. Undoubtedly, the VHAS survey and biomarker data bear the influences of these challenging climatic conditions and interpretation of the study results should take these conditions into account.

Finally, our extensive measures of past events during the wartime are reliant upon respondent recall of events that took place many decades prior. While memories may fade and recall may be biased, it is commonplace to rely upon aging study participants’ assessments of the past to characterize early life conditions. Several major studies of health and aging have unambiguously shown that survey questions about early life conditions can be successfully linked to later-life outcomes [52–54]. Many of our central measures of wartime stress, such as whether/how long an individual served in the military, whether a family member died during wartime, or whether they themselves killed others in the line of duty, should be subject to limited recall bias given the enormity of these events. The VHAS investigators remain cognizant and cautious as to the potential for recall error and subjectivity that may influence interview data. Accordingly, we have sought to triangulate information on past events, and to yield complementary biomarker and self-reported data on select health conditions as a means to assess validity.

## Data Availability

The dataset used in the current study are available from the corresponding author on reasonable request and with completion of data user agreement.

## Abbreviations

CAPI: Computer Assisted Personal Interviewing
CBC: Complete Blood Count
CHC: Commune Health Center
DNA: Deoxyribonucleic acid
EDTA: Ethylenediamine tetraacetic acid
HbA1c: Glycated Hemoglobin
PSU: Primary Sampling Unit
uL: Microliter
VHAS: Vietnam Health and Aging Study
